# Class IIa HDACs Are Important Signal Transducers with Unclear Enzymatic Activities

**DOI:** 10.3390/biom15081061

**Published:** 2025-07-22

**Authors:** Claudio Brancolini

**Affiliations:** Laboratory of Epigenomics, Department of Medicine, Università degli Studi di Udine, 33100 Udine, Italy; claudio.brancolini@uniud.it

**Keywords:** HDAC4, HDAC5, HDAC7, HDAC9, MEF2, enhancers, H3K27ac, H2BK120ac

## Abstract

Class IIa histone deacetylases (HDACs) are pleiotropic regulators of various differentiation pathways and adaptive responses. They form complexes with other co-repressors and can bind to DNA by interacting with selected transcription factors, with members of the Myocyte Enhancer Factor-2 (MEF2) family being the best characterized. A notable feature of class IIa HDACs is the substitution of tyrosine for histidine in the catalytic site, which has occurred over the course of evolution and has a profound effect on the efficiency of catalysis against acetyl-lysine. Another distinctive feature of this family of “pseudoenzymes” is the regulated nucleus–cytoplasm shuttling associated with several non-histone proteins that have been identified as potential substrates, including proteins localized in the cytosol. Within the complexity of class IIa HDACs, several aspects deserve further investigation. In the following, I will discuss some of the recent advances in our knowledge of class IIa HDACs.

## 1. Introduction

Acetylation of proteins is a widely used post-translation modification (PTM) that is exploited to influence a variety of cellular reactions. The amino group (NH_2_) attached to the ε-carbon of a lysine residue is the subject of such a PTM. Although the acetyl group is small, it can profoundly influence the protein surface by revoking the positive charge of the lysine. This can have a direct effect on the dynamics of local molecular interactions. In addition, acetylation of lysine can also maintain a local hydrophobic milieu that forms the binding site for the recruitment of proteins with specialized domains: the acetyl-lysine readers [1]. For these readers, the N-ε-acetylation of lysine represents a signal that is recognized by specific domains: the bromodomain, the YEATS domain, and the PHD fingers. These domains are characterized by the presence of a hydrophobic pocket suitable for the binding of peptide sequences containing an acetyl-lysine. These interactions modulate the assembly of multiprotein complexes on the DNA [2,3].

If the acetylated proteins are histones, this modification controls the accessibility to DNA and the assembly of multiprotein complexes on the chromatin, which activate gene transcription or modulate DNA repair [4]. The particular profile of histones acetylation can be maintained in the newly synthesized histones during DNA replication. Therefore, the memory of the differently accessible genome regions and the specific pattern of gene transcription can be inherited by the daughter cells after mitosis. This process is known as epigenetics [5,6].

**Figure 1:**
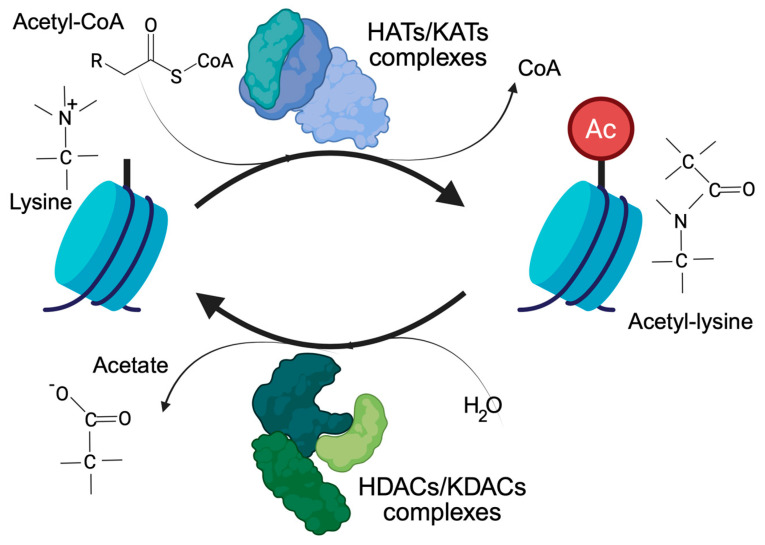
The control of lysine acetylation in histones by HATs/KATs (histone acetyl-transferase/lysine acetyl-transferase) and HDACs/KDACs (histone deacetylase/lysine deacetylase). Acetate can be converted back to acetyl-CoA by ACSS2. Only the ε-carbon group of lysine is shown.

Histone acetylation, and more generally lysine acetylation, is a dynamic and reversible process controlled by two antagonistic enzyme families, the KATs/HATs (lysine/histone acetyl transferases) and the KDACs/HDACs (lysine/histone deacetylases). KATs use the acetyl-CoA as a donor of the acetyl group. KDACs, on the other hand, produce acetate [7], which can be converted back into acetyl-CoA by the acyl-CoA synthetase short-chain family member 2 (Figure 1). Cells have different ways of supplying the nucleus with the acetyl-CoA required to regulate the accessibility of chromatin [2,8]. The link between acetylation, epigenetics, and metabolism is strong and clearly recognizable. Histone acetylation and gene transcription are energy-consuming processes and require metabolically active cells with available energy (ATP) and metabolic substrates. In view of the large number of histone proteins, histone acetylation also represents an important storage of acetyl groups within the cell [8].

## 2. An Overview of the Various Types of HDACs/KDACs

Eighteen KDACs/HDACs are present in humans, which are divided into zinc-dependent and NAD^+^-dependent enzymes. Five distinct classes of vertebrate HDACs have been proposed based on structural homologies to yeast HDACs and other features. The zinc-dependent enzymes form classes I, IIa, IIb, and IV. The NAD^+^-dependent enzymes are summarized in class III. Class I includes HDAC1, 2, 3, and 8. HDAC1 and HDAC2 often form common repressive complexes [7,9,10]. HDAC3 forms a distinct repressive complex with the co-repressors NCOR1 and NCOR2 (nuclear receptor co-repressor), which are required for the maturation of the catalytic activity of HDAC3 [11]. The assembly into multiprotein complexes supports the full activation of the catalytic activity of these three HDACs [10]. HDAC1/2/3 are important regulators of histone acetylation. HDAC8 differs from the other members of the class I family in that it exhibits strong catalytic activity as an isolated protein. However, HDAC8 is also subject to modulation as it possesses an allosteric domain that is structured in the form of a helix-loop-helix. This “allosteric domain” is the target of regulatory PTMs that modulate the activity of HDAC8 [12,13].

**Figure 2:**
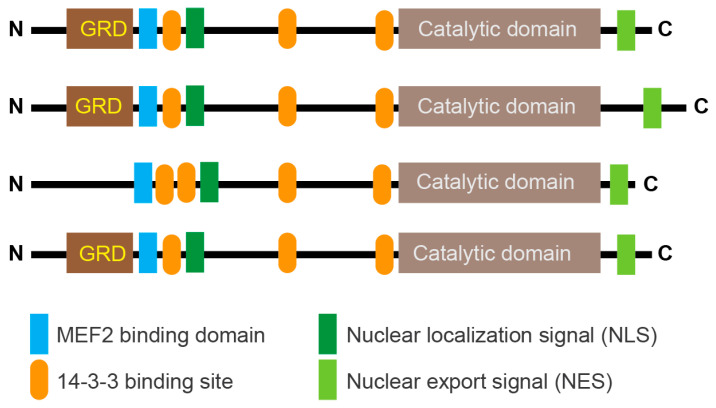
Schematic representation of the main domain organization in class IIa HDACs. Binding to 14-3-3 binding sites highlights the major serine residues that are phosphorylated by various kinases and promote nuclear export of deacetylases. In vertebrates, the deacetylase domain exhibits very low enzymatic activity due to the His/Tyr substitution in the catalytic site. The glutamine-rich domain (GRD) is indicated. The control of lysine acetylation in histones by HATs/KATs and HDACs/KDACs. Acetate can be converted back to acetyl-CoA by ACSS2. Only the ε-carbon group of lysine is shown.

Class IIa groups HDAC4, HDAC5, HDAC7, and HDAC9. These KDACs have an extended amino-terminal region that is designed to interact with multiple partners, including transcription factors such as members of the MEF2 family and co-repressors. This region contains an NLS (nuclear localization sequence) and phosphorylation sites recognized by 14-3-3 chaperones (Figure 2). The carboxy-terminal region contains the deacetylase domain and the NES (nuclear export sequence). In vertebrates, the tyrosine in the catalytic pocket is replaced by a histidine residue that renders the reaction inefficient against acetyl-lysine. This modification may have converted the class IIa HDACs into acetyl-lysine readers or may have altered substrate specificity.

However, alternative substrates for class IIa HDACs have not yet been clearly defined.

The activities of class IIa HDACs are tightly controlled by nuclear/cytoplasmic shuttling, which is monitored by the specific phosphorylation of the various 14-3-3 binding sites. Although the activities of class IIa HDACs are often controlled by their subcellular localization, the cells can use several additional mechanisms [14]. For example, proteasomal-mediated degradation can control the level of class IIa HDACs depending on phosphorylation [15,16,17]. Consequently, class IIa ubiquitylation can be used to modulate various cellular responses. Viral proteins can promote the degradation of HDAC4/HDAC5 to limit interferon production and enable viral infection [18]. Proteasomal degradation is also involved in switching off the repressive activity of HDAC4 during senescence [19].

Class IIb includes HDAC6 and HDAC10. HDAC6 contains two catalytically active domains, CD1 and CD2. The CD2 domain acts as an α-tubulin deacetylase and controls several other substrates [20]. The CD1 domain has very few substrates and shows specificity for C-terminal acetyl-lysine residues [21]. HDAC10 functions as a polyamine deacetylase and can protect cancer cells from chemotherapy by activating autophagy [22,23]. Class IIb are cytosolic enzymes with low activity towards acetylated histones [24].

The NAD^+^-dependent deacetylates are known as sirtuins, which in humans consist of 7 members (Sirt1/2/3/4/5/6 and 7). The sirtuins form the class III family of HDACs [25]. They are homologous to Sir2 (silent information regulator) in yeast and are found in various cellular compartments, including the mitochondria. Targets of sirtuins are histones as well as various non-histone proteins [26,27].

Finally, HDAC11 alone forms class IV. Some peculiarities of the catalytic domain of HDAC11 have justified the creation of this additional class of KDACs. This zinc-dependent deacetylase exhibits a higher enzymatic activity towards long-chain acyl groups, which has led to the definition of HDAC11 as a lysine de-fatty acylase [28]. Unicellular eukaryotes do not appear to possess class IV HDACs [29].

## 3. Class IIa HDACs

Class IIa HDACs are involved in several differentiation pathways that lead to the formation of various tissues, including blood vessels, bone, muscle, adipose tissue, myelination, and the immune system. These “pseudoenzymes” are also involved in the control of various adaptive responses, including cell proliferation, cell death, metabolism, migration, addiction, tissue injury, and many others [14,30,31,32,33,34,35,36,37].

Several reviews have addressed the contribution of class IIa HDACs to specific cellular responses, and it is difficult to find a specific context in which their contribution can be excluded. The versatility of class IIa HDACs is evidenced by various biological processes under their influence. It must also be considered that the expression of class IIa HDACs can be regulated by interconnected circuits based on transcription factors whose activities can also be regulated by the same class IIa HDACs [38]. This strategy guarantees compensation mechanisms between the different members of the family and can limit our knowledge of the contribution of an individual member to a specific cell function [39,40]. In addition, different class IIa HDACs can also cooperate to maintain a particular differentiation fate [41]. Another level of complexity is exemplified by HDAC9. A structural variant (SV) is present in the coding sequence of *HDAC9*. This SV affects the functionality of the *TWIST1* regulatory elements located within the *HDAC9* sequence. In fact, the 3′-*HDAC9* sequence functions as a critical *TWIST1* regulatory region that includes craniofacial *TWIST1* enhancers and CTCF sites. This SV is associated with craniosynostosis, a condition that alters postnatal skull and brain growth [42]. The complexity of class IIa HDACs can therefore also result from the inclusion of cis-regulatory elements that control the expression of other genes.

## 4. Structural Features of Class IIa HDACs

Although we have only partial information about the overall structure of class IIa, the definition of the structural characteristics of some specific domains/regions has provided insight into the possible mechanisms used by this gene family to modulate the various responses discussed here.

### 4.1. The N-Terminal Region

As shown in Figure 2, the N-terminal region of HDAC4/5 and 9 contains a glutamine-rich domain (GRD). Structural studies with the carboxy-terminal part of the HDAC4 GRD have shown that it can fold into a long helix that is dynamically balanced between dimer and tetramer [43]. This domain may also be involved in hetero-oligomerisation between the different members of the class IIa HDACs family. Recently, Dai et al. co-crystallized a complex between human HDAC4-GRD (aa 62–192) and the MADS box/MEF2s domain of MEF2A (aa 1–95) in the presence of a 15-mer DNA duplex with the MEF2 consensus sequence [44].

**Figure 3:**
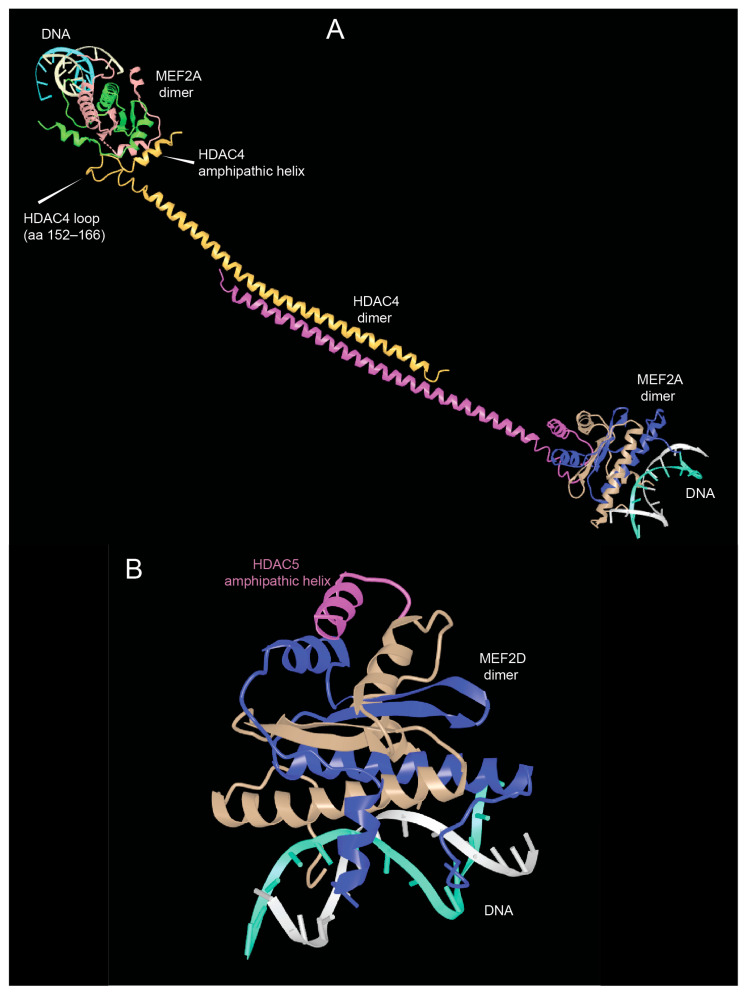
The domains of the amino-terminal region of class IIa HDACs. (**A**) View of the glutamine-rich domain (GRD) of HDAC4 in complex with MEF2A. PDB ID: 7XUZ https://www.ncbi.nlm.nih.gov/Structure/icn3d/full.html?&mmdbid=239077&bu=0&showanno=1&source=full-feature, 3 June 2025. (**B**) Crystal structure of the MADS box/MEF2 domain of MEF2D bound to dsDNA and the HDAC5 deacetylase peptide containing the MEF2 binding motif. PDB ID 8Q9P. https://www.ncbi.nlm.nih.gov/Structure/icn3d/full.html?&mmdbid=245462&bu=0&showanno=1&source=full-feature, 3 June 2025. Each MEF2 dimer is shown with different colors (brown/blue and brown/green)—HDAC4-GDR monomer is shown with different colors (pink/yellow).

The analyzed region of HDAC4 consists of an amphipathic helix that binds MEF2 (see below), which exits the GRD after a loop. This loop also contributes to binding with MEF2A. In this new structure, the GRD is folded into an elongated helix, a conformational change possibly favored by MEF2A binding. The GRD enables the dimerization of HDAC4 and the coordination of two different MEF2A-DNA complexes (Figure 3A).

The formation of the complex between MEF2A and HDAC4 could also influence the ability of HDAC4-GRD to tetramerize and form higher order structures that favor the stabilization of dimers [43]. In addition, HDAC4-mediated dimerization of HDAC–MEF2A complexes may bridge to distant DNA sites to create a local repressive chromatin environment in a more productive manner [44].

Additional structural studies using class IIa HDACs peptides corresponding to the amphipathic helices that bind MEF2D have shown that these peptides are unstructured in solution and adopt a folded α-helical structure only after binding to MEF2D (Figure 3B). Furthermore, the adaptability of the hydrophobic MEF2 furrow that accommodates these class IIa peptides explains the multiple protein–protein interactions involving other transcriptional regulators that are recognized by MEF2 via this domain [45].

### 4.2. The Catalytic Domain

**Figure 4:**
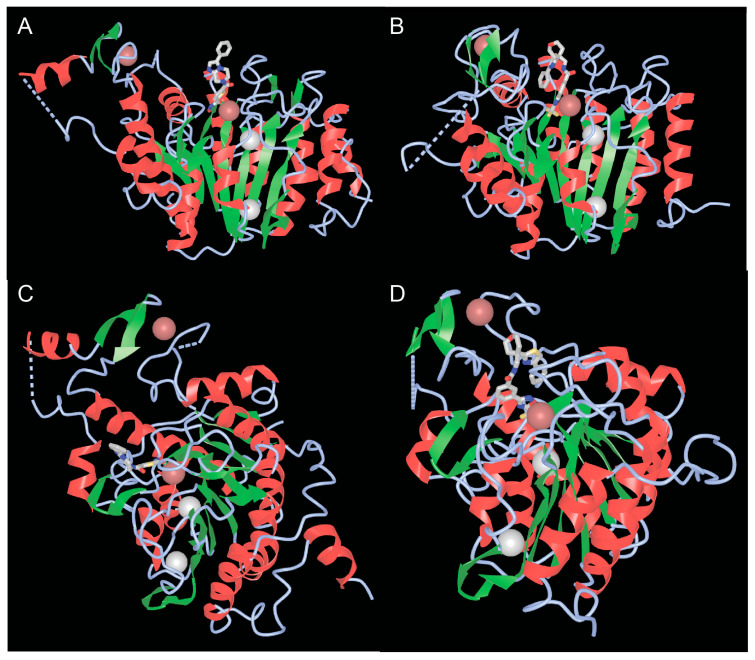
The catalytic domains of class IIa HDACs. (**A**,**B**) View of the catalytic domains of HDAC4 (**A**) and HDAC7 (**B**) showing the catalytic pocket with the coordinated zinc and adjacent potassium ions. (**C**,**D**) Rotated view of the class IIa catalytic domain highlighting the second structural zinc ion and the structural zinc-binding domain (sZBD) of HDAC4 (**C**) and HDAC7 (**D**). PDB ID 2VQM. https://www.ncbi.nlm.nih.gov/Structure/icn3d/full.html?&mmdbid=65359&bu=1&show-199%20anno=1&source=full-feature, 3 June 2025. The α-helices are shown in red and β-strands in green.

Well-defined functional activities embedded in the carboxy-terminal region of class IIa are the catalytic domain and the NES. The catalytic domain is structurally conserved between the four members [33,46]. The structure of the catalytic domains of HDAC4 and HDAC7, whether in complex with different inhibitors or not, has been solved [47,48]. These studies have revealed the homologies with the corresponding domain of class I HDACs, characterized by a central parallel β-sheet with eight β-strands (Figure 4A,B). In addition to the zinc ion, which is located within the catalytic domain, there are two potassium ions in the vicinity of the active sites, similar to class I HDACs (Figure 4A,B).

The low class IIa deacetylase activity in vertebrates depends on the substitution of tyrosine 976 (in HDAC4, Y843 in HDAC7) by a histidine in the catalytic pocket. This tyrosine is considered important for stabilizing the transition state by hydrogen bonding with the oxyanion intermediate, whereas the histidine, which is turned away from the active site (out conformation), makes the transition state less stable and deacetylation less efficient [47,48,49,50]. However, binding of some protein partners or substrates of class IIa HDACs (including sequences in addition to acetylated lysine) could promote inward rotation of the histidine side chain, orienting the histidine ring towards the catalytic site and the acetyl group of the substrate. An “in” conformation that could support catalysis involving a water molecule [47,48]. Recently, analyses of different hDAC4 crystal structures and computer simulations have shown that a rotational transition between the “in” and “out” conformation of H976 is possible due to the very high flexibility of the loop segments around this residue [51].

Another special feature of the catalytic domain of class IIa HDACs is the lack of a water-filled tunnel. In class I HDACs, this tunnel should allow the release of the acetate reaction product [47].

Although class IIa enzymatic activity can be measured on a non-physiological substrate (the trifluoroacetyl-lysine), a detailed study using a peptide library was performed to determine the substrate specificities of class IIa HDACs. With respect to trifluoroacetyl-lysine, the aa were mapped at positions −3 and +3. Interestingly, class IIa HDACs show a strong preference for bulky aromatic acids flanking the central trifluoroacetyl-lysine, while positively charged residues and proline are not favored [52]. These results could help to further improve the development of specific class IIa inhibitors.

The question of possible alternative class IIa substrates has not yet been resolved. Recently, de-crotonylation activity has been associated with HDAC7. During leucine deprivation-induced autophagy, 14-3-3 proteins are crotonylated. This PTM reduces interactions with partners, including protein phosphatase 1B (PPM1B), which stimulates autophagy via ULK1. Specific class IIa inhibitors increase the crotonylation of PPM1B [53]. However, further studies are needed to determine whether HDAC7 can function as decrotonylases or whether this activity is mediated by an associated class I HDAC [54].

### 4.3. The Second Structural Zinc Ion

A third important feature of the catalytic domain of class IIa HDACs is the presence of a second zinc ion that fulfills structural roles and is also of interest as a potential target for selective class IIa inhibitors (Figure 4C,D). The structural zinc ion is crucial for the connection of two segments of HDAC4. The first segment is a 17 amino acid loop (Lα1-α2) containing three residues (His665, Cys667, and His678), which chelate zinc. The other segment is a helix-turn-helix motif of 35 amino acids (α6-α7-β3-β4), followed by a β-hairpin with the fourth residue that can chelate zinc (Cys751). This domain is known as the structural zinc-binding domain (sZBD).

By comparing the inhibitor-free and inhibitor-bound structures of the catalytic domain of HDAC4, Bottomley and co-authors proposed that the inhibitor can influence the conformation of the sZBD. An open conformation is stabilized by the inhibitor, while the closed conformation is the native conformation. The authors concluded that different conformations of the sZBD may be required to allow conformational flexibility and may play a role in regulating HDAC4 function. Indeed, this structural domain in the closed conformation is also involved in regulating the interaction with the repressive complex HDAC3-NCOR1/NCOR2 [47].

Recent studies have shown that the sZBD plays a key role in the global structural integrity and stability of HDAC4, but these studies have also challenged the open conformation hypothesis and questioned its existence. Therefore, the authors suggest that only the closed conformation should be considered for the development of HDACs-specific class IIa-inhibitors [55].

### 4.4. NCOR1 and NCOR2

NCOR (nuclear receptor co-repressor) 1 and 2, the latter originally called SMRT (silencing mediator of retinoic acid and thyroid hormone receptor), are pleiotropic transcriptional co-regulators. Their activities, which are not redundant, are crucial for mouse development [56,57,58]. Although the multiprotein complexes that assemble with NCOR1 and 2 change in different contexts, some partners are stably observed. These include the following: HDAC3, which is the major partner and requires NCORs to exert full enzymatic activity; the G protein pathway suppressor (GPS2), the transducing β-like 1 (TBL1) and its homolog, TBL-related 1 (TBLR1). Under certain conditions, the NCOR complex can also associate with HDAC1 [59,60]. NCOR proteins can be considered as large platforms with an average weight of about 270 kDa that support interaction with multiple partners. Different NCOR domains mediate interactions with specific partners. At the amino terminus, the RDs (repressive domains), which are highly conserved between NCOR1 and 2, mediate interaction with GPS2 and TBL1 or other co-repressors, including class IIa HDACs recognized by RD3. The SANT-like domains are important for deacetylation and one of them includes the deacetylase activation domain (DAD), which binds HDAC3. One of the SANT domains is part of the deacetylase activation domain (DAD). Finally, there are 3 receptor interaction domains (RIDs) that mediate the interaction with nuclear receptors [58].

A conserved repetition of eight amino acid motifs with the consensus sequence G-S-I-t/s-q-G-t-P characterizes RD3. In particular, the repetition of the GSI motif was hypothesized to be critical for the interaction with class IIa HDACs. The GSITQGTP corresponding to sequence 1450-1469 of NCOR2 is sufficient to bind the catalytic domain of class IIa HDACs. Within this sequence, the GSI and the G and T at position 6 and 7, respectively, are absolutely required for the interaction. On the other hand, mutations of critical zinc chelating residues within the sZBD of HDAC4 have shown that the peptide and thus the NCORs recognize these surface loops near the catalytic site and in closed conformation [61].

### 4.5. The Nuclear Export Signal (NES)

The regulation of class IIa HDACs nuclear export plays a central role in various cellular states from cell proliferation/survival to differentiation and in many adaptive responses [14,25,31,32,33,34,62,63].

The export of proteins from the nucleus is often mediated by a leucine-rich NES sequence. In general, this sequence is a short stretch of 8–15 amino acids with regularly spaced hydrophobic residues. NESs are the consensus sequence recognized and bound by the export karyopherin [64]. Many of the identified leucine-rich NESs deviate significantly from the generally accepted loose consensus L-x(2,3)-[LIVFM]-x(2,3)-L-x-[LI] [65]. This has led to the classification of NES into different subclasses [66]. An NES binds to the hydrophobic pocket (labeled P0–P4) in the hydrophobic groove defined by the HEAT repeats 11 and 12 of CRM1/Exportin-1 [66,67].

**Figure 5:**
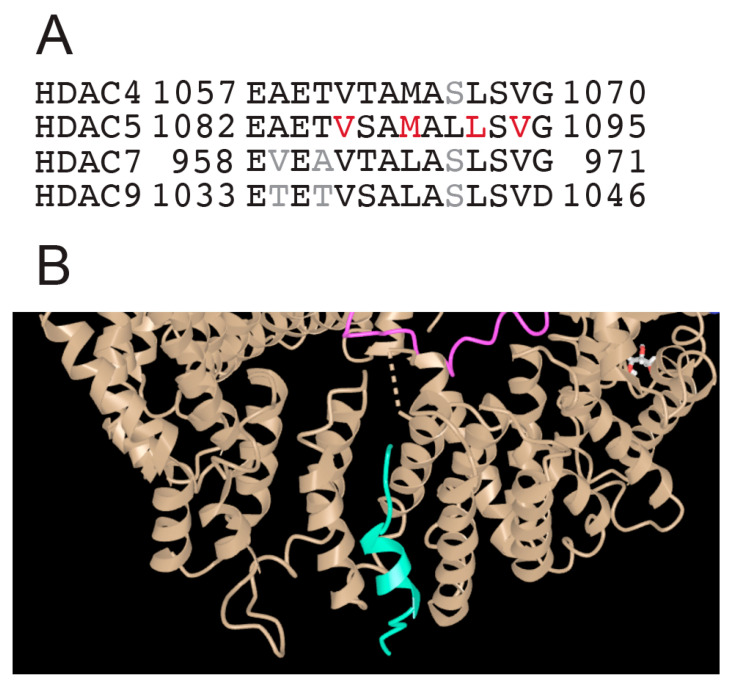
The NES of class IIa HDACs. (**A**) Alignment of the class IIa carboxy-terminal sequence with the different nuclear export signals (NESs). (**B**) Enlarged view of the structure of the HDAC5 NES peptide (green) in complex with CRM1/Exportin-1 (brown). The original molecular complex also contains RAN (pink) and RAN-GAP (blue). PDB-ID 5UWI. https://www.ncbi.nlm.nih.gov/Structure/icn3d/full.html?&mmdbid=148948&bu=1&showanno=1&source=full-feature, 3 June 2025.

At the carboxy terminus, after the deacetylase domain, all class IIa HDACs contain an NES. This sequence is highly conserved among the different members, with the only difference that in HDAC4/5 a methionine has been replaced by a leucine, in HDAC7/9 as the third key hydrophobic residue of the motif (Figure 5A). In these sequences, the positions of the hydrophobic residues do not indicate the formation of an amphipathic helix. However, the corresponding peptide of HDAC5, which was used to define the complex with CRM1/exportin-1, partially folds into an α-helix (Figure 5B) [68].

Phosphorylation of 14-3-3 binding sites affects NES activity, and an integral NES is required for the export of class IIa HDACs even in the presence of 14-3-3 binding [69,70,71]. In the case of HDAC7, the forced expression of CRM1 can trigger the nuclear export of deacetylase even in the presence of mutations at the 14-3-3 binding sites [72].

## 5. Class IIa HDACs as Pleiotropic Regulators of Both Differentiating and Adaptive Responses

Class IIa HDACs are considered pleiotropic genes that are involved in the regulation of a broad spectrum of biological responses. One example discussed here is HDAC4.

In the posterior hypothalamus, Hdac4 and Hdac5 regulate non-rapid eye movement sleep (NREMS). Phosphorylation of Hdac4 and Hdac5, which occurs via the Lkb1-Sik3 signaling pathway, is associated with an increased need for sleep, and *Hdac4* haploinsufficiency in mice increases sleep [73,74]. In humans, both haploinsufficiency of *HDAC4* and point mutations that impair binding to 14-3-3 proteins are associated with sleep disorders. These point mutations in HDAC4 are responsible for several additional alterations, including dysmorphic facial features, dysphagia and/or drooling, congenital hip dislocation, and progressive kyphoscoliosis [75]. Haploinsufficiency of *HDAC4*, on the other hand, is responsible for 2q37 deletion syndrome, a disorder characterized by brachydactyly type E (BDE) and typical facial features. The haploinsufficiency is not completely penetrant and additional defects vary in severity between patients [33,76,77].

HDAC4 is abundantly expressed in bone and cartilage, and osteoblast lineage-specific knock-outs have demonstrated its key role in skeletal growth. The most obvious defects in these mice are premature ossification of developing bone [78,79]. In particular, conditional knock-out of *Hdac4* in osteoprogenitors is associated with increased susceptibility to dwarfism, growth plate closure, and osteoporosis progression [79].

Further studies in mice in which *Hdac4* was silenced in a tissue-specific manner have revealed a contribution of deacetylase in various contexts, including the mediation of inflammatory pain [80], proliferation and differentiation of satellite cells [81] or protection of the diabetic heart [82]. It has also been reported that the cytoplasmic functions of HDAC4 are critical for plasma membrane repair in a mouse model of Duchenne muscular dystrophy [83].

In summary, genetic studies and animal models have clearly demonstrated the involvement of HDAC4 in a variety of cellular responses that require modulation of gene expression and readjustment of epigenetic profiles. However, epigenetically independent effects of HDAC4 have also been observed. The example of HDAC4 briefly discussed here shows the pleiotropic nature of class IIa HDACs, which is probably underestimated given the redundancy and compensatory circuits that characterize the regulation of this enzyme family.

## 6. Recent Advances in Class IIa HDACs Biology

The contribution of class IIa HDACs to various differentiation and adaptation responses has been discussed in many reviews [14,31,32,33,34,62,63]. Therefore, in the following sections I will focus on the new results that have been published in recent years.

### 6.1. DNA Repair

The contribution of class IIa to DNA repair was initially investigated in a few studies, but only recently have details of the mechanisms involved become known [84,85,86]. Repair of DNA double-strand breaks (DSBs) can occur mainly via two pathways: non-homologous end joining and homologous recombination. Alternative native end-joining repair mechanisms can also be used [87].

HDAC4 may act as an epigenetic regulator of H2BK120 acetylation at the sites of DSBs through a complex with HDAC1/HDAC2. This activity is important for the proper recruitment of DNA repair enzymes, including BRCA1, which is required for homology-directed repair [88]. Interestingly, class IIa HDACs can also repress the expression of genes involved in non-homologous end joining in an MEF2-dependent manner [89].

Additionally, class IIa HDACs can influence DNA repair by controlling the acetylation of non-histone proteins. HDAC5 can deacetylate PARP1 at Lys498 and Lys521. Acetylation of these residues limits PARP1 PARylation and DNA repair [90]. As a result, depletion of HDAC4 and HDAC5 increases the amount of unrepaired DNA, as indicated by yH2AX positivity. HDAC4 and HDAC5 may also indirectly contribute to DNA repair by downregulating gene transcription through acetylation of ATF9, a component of the elongation machinery. The authors propose that the export of class IIa HDACs to the cytoplasm upon DNA damage, as driven by CamKII, favors acetylation of ATF9 [91]. For both examples of PARP1 and ATF9 deacetylation, it remains to be clarified whether class I HDACs in complex with HDAC5 can contribute to enzymatic activity.

### 6.2. Senescence

It has recently been shown that class IIa HDAC can inhibit senescence in certain cellular models [19,92,93,94]. Since senescence can also be triggered by the accumulation of DNA damage [95,96,97,98], the influence of class IIa HDACs on the efficiency of DNA repair and the accumulation of DNA damage could be the cause of the senescent phenotype. Certainly, the transcriptional control exerted by class IIa can also help to antagonize the epigenetic and transcriptomic profiles that characterize senescence, particularly through the control of super-enhancers [19,92,93].

### 6.3. Metabolism

Class IIa HDACs are known substrates of SIKs (Salt-inducible kinases) that control their nuclear export [14]. In a brown mouse cell line, Hdac4, but not other class IIa HDACs, is required for the expression of the key factor for thermogenesis, uncoupling protein 1 (Ucp1). The activity of Hdac4 is explained by the deacetylation and activation of PGC1α (peroxisome proliferator-activated receptor gamma (PPARγ) co-activator-1α), an important co-activator of gene expression [99]. Mitochondrial metabolism is also influenced by HDAC7, but in a different context and in a different way. In renal cell carcinoma, HDAC7 suppresses the expression of enzymes involved in the tricarboxylic acid cycle (TCA). In this malignant disease, HDAC7 is activated by TGF-β signaling to alter TCA metabolism [100].

### 6.4. Class IIa HDACs and Differentiation

The effect of class IIa on epigenome rearrangement can be dramatic. During terminal erythroid differentiation, chromatin condensation is followed by nuclear polarization and extrusion of the condensed nucleus [101]. In an in vitro model of erythroid differentiation, HDAC5 expression is upregulated, and its knockdown impairs differentiation, nuclear condensation, and enucleation. This effect correlates with a specific defect in H4K12 acetylation, which is not reduced during erythroid terminal differentiation in the absence of HDAC5 [102].

In other contexts, single class IIa affects the epigenome mainly on regions subject to dynamic regulation and buffers the acetylation level of accessible regions. It is less clear whether class IIa may play a role in maintaining the heterochromatic regions of the genome [102,103,104,105]. Whether the simultaneous removal of the different family members could affect the opening of heterochromatic regions is an open question that deserves further investigation.

The role of class IIa in specific differentiation pathways could imply a radical change in the epigenetic status of cells to allow transcription of differentiation genes and repression of unnecessary genes. There are several examples of differentiation pathways regulated by class IIa [14,31,32,33,34,62,63].

Recently, a correlative analysis has pointed to the possible role of Mef2a and Hdac9 in determining a subpopulation of olfactory sensory neurons in the mouse [106]. Phosphorylation of 14-3-3 binding motifs is associated with the reprogramming of fibroblasts into cardiomyocyte-like cells. Various TFs are involved in this reprogramming, including Mef2c. Phosphorylation of 14-3-3 binding motifs in Hdac4 resolves nuclear condensates, and the phosphatase PP2A is important for controlling phosphorylation turnover and these condensates define transcriptionally repressed regions. Disruption of Hdac4 condensates stimulates cardiac reprogramming [107]. The observation of HDAC4 as nuclear speckles/foci/aggregates/condensates has also been reported in previous studies, particularly when analyzing the localization of ectopically expressed proteins mutated at 14-3-3 binding sites [15,85,108]. In principle, the formation of these HDAC4 condensates could depend on the GRD, but the class IIa-specific inhibitor TMP269 was sufficient to dissociate Hdac4 condensates in induced cardiomyocyte-like cells, suggesting the existence of some alternative mechanisms [107].

In epithelial cells, HDAC7 represses genes involved in cell junction assembly and membrane organization—an activity that alters tissue architecture by inhibiting cell polarity, cell differentiation, and the formation of primary cilia and lumen [109]. It is assumed that the activity of HDAC7 is regulated by the transmembrane protein complex Crumbs/PATJ/Lin-7 [110]. Whether the inhibition occurs through sequestration of HDAC7 into the PATJ protein complex requires further investigation.

From these few examples it is clear that the role of class IIa in differentiation is pleiotropic, sometimes antagonistic, but in other cases also promoting differentiation, and that the cells use several mechanisms to regulate their activities.

### 6.5. Differentiation and the Immune System

HDAC7 has several functions in the differentiation of immune cells. It is required for the development of both T cells and B cells and controls the differentiation and activation of lymphocytes as well as inflammatory signaling in macrophages [32,111,112,113,114,115]. This complexity can also be regulated by alternative splicing [116]. Recent advances have drawn attention to the contribution of HDAC7 in controlling the final stages of effector T cell differentiation. HDAC7 is stabilized by phosphorylation of salt-inducible kinase 1 (SIK1), accumulates in cell nuclei, where it reduces histone 3-lysine 27 acetylation (H3K27ac) at specific cytokine loci, thereby reducing their expression [117]. The effects of SIKs on the class IIa nuclear/cytoplasmic shuttling, with some lineage dependent peculiarities, have been documented in several studies [63,118,119]. Normally, class IIa phosphorylation by SIKs is associated with nuclear export. Indeed, SIK1 controls HDAC7 in cardiomyocytes by promoting its stabilization and cytoplasmic localization. In contrast to the other class IIa HDACs, here, HDAC7 promotes cardiac hypertrophy by controlling c-Myc expression [120].

In the context of T cell subpopulations, Hdac4 and Hdac7 can modulate the differentiation of Th17 cells. These cells are a subset of pro-inflammatory CD4+ helper T cells [121]. Hdac4 and Hdac7 are upregulated during Th17 differentiation and are highly expressed compared to other T cell subsets (Th1, Th2 and Treg). The promoters of Hdac4 and Hdac7 are occupied by Th17 lineage-specific TFs and H3K27ac is increased. What is special about this differentiation is the role of Hdac4, which promotes the transcription of lineage-specific genes via an interaction with JunB. In contrast, Hdac7 in complex with Aiolos and the co-repressors Ncor1/Ncor2/Hdac3 represses the non-lineage-specific genetic programs [41]. Previous studies have provided evidence for the complexity of the class II contribution in T cells. Hdac5 is required for the efficient generation of T regulatory (Treg) cells and also for the full activity of CD8(+) T cells [122]. In Treg cells, Hdac9 controls the acquisition of an effector phenotype by repressing Mef2d transcriptional activity [123].

In the differentiation of B cells, Hdac7 plays an important role in the transition from the pro-B to the pre-B cell stage. Deletion of Hdac7 leads to chromatin decondensation and changes in gene expression. The genes suppressed by Hdac7 include the enzyme ten-eleven translocation 2 (TET), which triggers DNA demethylation by converting 5-methylcytosine (5-mC) into 5-hydroxymethylcytosine (5-hmC). Hdac7 binds to both the promoter and an enhancer that controls *Tet* transcription. Changes in 5-hmC lead to aberrant transcription of miRNAs and transposable elements LINE-1 [115]. An effect on repetitive elements was also observed for HDAC4 during senescence, leading to an interferon response due to the accumulation of dsRNAs [94].

### 6.6. Class IIa HDACs and Inflammation

There are several indications of a contribution of HDAC9 to the development of cardiovascular disease (CVD) and the formation of atherosclerotic plaques [34]. Genetic variants in the HDAC9 locus are associated with stroke, myocardial infarction or CVD [34,124,125]. These variants are located within cis-regulatory elements (CREs) that control the expression of HDAC9 itself and possibly other genes. The contribution of HDAC9 to the development of vascular diseases has been investigated and several pathological mechanisms have been observed and proposed. HDAC9 may induce a dysregulated inflammatory response in both macrophages and endothelial cells by controlling the acetylation and activation of IKK (inhibitory kappa B kinase)-α and β [126]. HDAC9 may also be involved in endothelial–mesenchymal transition, a feature associated with CVD [127].

A mouse model was developed in which the CRE variant rs2107595, which regulates HDAC9 expression and is associated with chronic inflammation and atherosclerosis, was deleted [128]. Deletion of this CRE leads to upregulation of Hdac9 expression in a cell lineage-specific manner. Hdac9 was upregulated in bone marrow-derived macrophages and myeloid cells, but not in T cells, smooth muscle cells, and endothelial cells. By transplanting cells, the authors were able to show that the upregulation of Hdac9 in myeloid cells exacerbates atherosclerosis and worsens plaque destabilization. This effect is associated with an increased production of inflammatory cytokines. A direct influence of HDAC9 on the inflammasome and the activation of Caspase-1 is suspected as a mechanism [128]. Inflammasomes are cytosolic multiprotein complexes that recognize pathogen-associated molecular patterns (PAMPs) and damage-associated molecular patterns (DAMPs) to initiate inflammatory responses [129]. NOD-like receptor pyrin domain-containing 3 (NLRP3) is a member of the major family of receptors involved in inflammasome activation. HDAC9 interacts with NLRP3 and mediates its deacetylation, which promotes oligomerization and activation of Caspase-1. Caspase-1 can then process Gasdermin D (GSDMD) to initiate pyroptosis [128]. These data point to a new function of HDAC9 that may also have profound implications for CVD from a therapeutic perspective. However, there are still some open questions, especially related to the very low catalytic activity of HDAC9. Are class I HDACs in complex with HDAC9 involved in this process? The molecular details of the interaction between HDAC9 and NLRP3 also require further investigation. In addition, the link between class IIa HDACs and pyroptosis may be even more complicated. Another study suggested that HDAC4 directly deacetylates GSDMD, specifically at lysine 248, which acts as an inhibitor of pyroptosis [130]. This study also needs to better define whether associated class I HDACs contribute to the enzymatic activity proposed for HDAC4.

### 6.7. Functions in the Central Nervous System (CNS)

In addition to the sleep modulation described above, class IIa HDACs are involved in various adaptive responses in the CNS. Interest in Hdac5 was sparked by its role in cocaine-induced behaviors [131,132]. In the nucleus accumbens, Hdac5 also limits relapse-associated behavior after operant heroin self-administration and forced abstinence [133]. Hdac5 activity is described in two distinct populations of medium spiny neurons that suppress cue-induced heroin craving and heroin-induced drug craving. Hdac5 acts epigenetically to influence neuronal excitability, thereby reducing the formation of strong, relapse-inducing drug memories [133]. The importance of HDAC5 in the regulation of brain functions and plasticity finds important confirmation from an evolutionary perspective. A search for human-specific deletions in conserved regions (hCONDELs) compared to the chimpanzee genome identified a single base deletion in an active enhancer of HDAC5. It is hypothesized that this deletion restricts the expression of HDAC5. A new condition that could affect neurogenesis in the human brain [134] and that was confirmed by preliminary experimental data [135].

## 7. The Genomic Landscape Under the Influence of Class IIa HDACs

Although class IIa can also affect the acetylation of non-histone proteins, their effect as epigenetic regulators has been observed in several studies and further strengthened by defining their binding to specific genomic regions [19,41,103,104,105,131,136,137,138]. There are three main strategies by which class IIa can bind to DNA. First, they can be recruited as partners of selected TFs that confer the DNA-binding motif. An example is represented by the MEF2 family of TFs, well-known partners of class IIa HDACs [139]. Alternatively, they could be recruited by other epigenetic regulators that contain subunits that act as readers of histone PTMs. The third possibility is that the class IIa HDACs themselves act as readers of acetyl-lysine via the “pseudocatalytic” domain and serve as epigenetic regulators via the positioning of the class I HDACs. In fact, the dissociation constants of class IIa HDACs for acetyl-lysine are within the range of binding affinities observed for bromodomains (Kd = 10–100 μm) [140,141,142]. In addition, in vitro studies have shown that binding to acetylated peptides can affect the interaction with NCOR1 and thus the associated deacetylase activity of HDAC3 [142]. Although this study is limited by the use of ectopically overexpressed proteins, it is conceivable in principle that once class IIa HDACs recognize an acetylated histone, disassembly of the NCORs/HDAC3 complexes could allow deacetylation of the surrounding nucleosomes, leaving the nucleosome bound by class IIa protected from deacetylation. A small region of acetylated histone could represent some sort of memory of a previous acetylated region. Of course, further studies are needed to prove this hypothesis.

However, FRAP experiments indicate that class IIa shows a highly dynamic interaction with chromatin with high on/off rates [71]. There is an indication of highly transient interactions with DNA as a possible consequence of the various PTMs, mainly phosphorylations, controlling the repressive activities of class IIa HDACs. An alternative hypothesis could consider the involvement of an associated deacetylase activity that removes the acetyl group shortly after the class IIa HDACs recognize an acetyl-lysine. On the other hand, ChIP-seq experiments have shown that class IIa HDACs can accumulate in specific genomic regions [19,41,103,104,105,131,136,137,138]. It is noteworthy that these regions are acetylated regions which are often analyzed for H3K27ac. This observation suggests that class IIa may be part of an epigenetic sensor that constantly acts on regions that are highly dynamic in terms of acetylation status and gene transcription. If the expression of class IIa HDACs is disrupted, this affects the acetylation level of these bound regions and the expression of the genes they regulate.

The influence of class IIa on histones is not limited to the control of acetylation status. Other PTMs can also be regulated to achieve a more stable definition of chromatin state. In a study aimed at exploring the contribution of alternative splicing of HDAC7 to epigenetic control, mass spectrometry studies have demonstrated the effect of HDAC7 not only on H3K4ac and H3K76ac, but also on the methylation status of H3K27 and H3K36 residues [116], a regulation that is important for controlling the expression of T cell surface markers [116]. In cardiomyocytes, HDAC4 can also act as a scaffold for regulated histone methyl-transferase activities to complete a repressive signature, as in the case of H3K9m3, which can be subverted by cardiac preload and HDAC4-induced nuclear export [143].

The activity of class IIa HDACs as triggers/supervisors of various histone PTMs to repress gene transcription has also been reported in other studies, although the molecular complexes involved are not clearly defined. In cardiomyocytes, deletion of HDAC4 promotes the acquisition of an open chromatin state at cardiomyocyte-related genes defined by histone marks of active transcription (H3K4me3, H3K9ac, and H3K27ac) around the relative TSS. These regions are characterized by the presence of MEF2 binding sites [144].

Surprisingly, however, the corresponding genes were not upregulated in cells in which HDAC4 was knocked out. These promoters are poised. Physical exercise activates the binding of MEF2, which removes H3K9me2-mediated repression and allows gene transcription to occur.

## 8. Conclusions

The ability of class IIa HDACs to modulate so many different biological processes is remarkable. This complexity is compounded by the alteration of the catalytic site, which no longer effectively triggers the deacetylation of lysine residues. These changes, which have occurred during vertebrate evolution, implicate the ability of class IIa to recruit other HDACs. This has led to the evolution of class IIa as a platform for the coordination of multiprotein complexes, which may explain some of these pleiotropic effects. To understand the mechanisms employed by class IIa in different contexts, it is important to define the molecular composition of these complexes. A challenge that requires intensive studies as well as better reagents and most likely more accurate cellular models that come closer to physiological in vivo conditions. Sophisticated mass spectrometric approaches should be used to dissect the class IIa HDACs-specific interactomes and clearly distinguish between true partners and contaminants [88,130,145].

In recent years, many biological functions have been characterized under the supervision of class IIa. Now it is time to better understand how class IIa HDACs work.

## Abbreviations

The following abbreviations are used in this manuscript:

CRE	Cis-regulatory elements
CVD	Cardiovascular disease
GRD	Glutamine-rich domain
HDAC	Histone deacetylase
HAT	Histone-acetyl transferase
KAT	Lysine-acetyl transferase
MEF2	Myocyte Enhancer Factor
NCOR	Nuclear receptor co-repressor
NES	Nuclear export signal
NLS	Nuclear localization signal
PTM	Post translational modification
sZBD	structural zinc-binding domain
